# Risk of osteoporotic fracture in women using the FRAX tool with and without bone mineral density score in patients followed at a tertiary outpatient clinic ‒ An observational study

**DOI:** 10.1016/j.clinsp.2022.100015

**Published:** 2022-03-12

**Authors:** Maria Helena Sampaio Favarato, Maria Flora de Almeida, Arnaldo Lichtenstein, Milton de Arruda Martins, Mario Ferreira Junior

**Affiliations:** Serviço de Clínica Geral e Propedêutica, Hospital das Clínicas, Faculdade de Medicina, Universidade de São Paulo (HCFMUSP), São Paulo, SP, Brazil

**Keywords:** Osteoporosis, Screening, Multimorbidity

## Abstract

**Objectives:**

Fragility fractures increase morbidity and mortality. Adding assessment of clinical risk factors independently or as a previous step to Bone Densitometry (BD) should provide better accuracy in fracture risk prediction. FRAX tool might be used to stratify patients in order to rationalize the need for BD and risk classification. The primary objective of this study is to describe and perform comparisons between the estimated risk of fractures in 10 years using the FRAX calculator based on clinical factors with and without BD results for women aged 40 or more with clinical diseases monitored in tertiary care service in internal medicine.

**Methods:**

Cross-sectional. Women over 40 years with BD in the previous year. After medical chart review, identification of risk factors and risk estimations using FRAX-BRAZIL with (FRAX BDI) and without (FRAX BDNI) the inclusion of T-score.

**Results:**

239 women. Age 65 ± 10.35 years. BMI 29.68 ± 6.27kg/m^2^. Risk factors: 32(13.4%) previous fractures; 23 (9.6%) current smoking; 78 (32.6%) corticosteroids use; 44 (18.4%) rheumatoid arthritis; 38 (15.9%) secondary causes; FRAX scores were higher when BD was not included. Spearman correlation coefficients between FRAX BDNI and FRAX BDI for major fractures *r* = 0.793 (95% CI 0.7388‒0.836). For hip fractures *r* = 0.6922 (95% CI 0.6174‒0.75446)

**Conclusion:**

Using FRAX to estimate 10-year fracture risk without BD data might be a reliable tool for screening, even for patients with a high prevalence of risk factors, improving accessibility and equity in health systems. The present study's data suggest an overestimation of fracture risk with FRAX BDNI, suggesting that it is safe to be widely used as a screening tool.

## Highlights

• FRAX without BD moderately to strongly correlates to FRAX with BD and tends to superestime 10-year fracture risk prediction.
• FRAX without BD is reliable and sensitive as a screening tool even in multimorbid patients.

## Background

Fragility fractures are important factors of morbidity and mortality. The susceptibility for such fractures comes through the interaction of clinical and epidemiological factors with bone mineral density.^1^ In spite of the recommendations of several international scientific entities that consider age as the most important risk factor,^2^, ^3^, ^4^ there is no universally accepted policy for population-based screening of osteoporosis. Using densitometry data provides specific but not sensitive information, with most fractures occurring in people with T-scores bigger than -2.5. Thus, adding assessment of clinical risk factors independently or as a previous step to bone mineral densitometry should provide better accuracy in fracture risk prediction.^5^

The FRAX tool has been developed by the Sheffield University in collaboration with the World Health Organization, and has been validated in different countries, taking into account local studies and epidemiological data to substantiate its clinical applicability in different populations.^1^^,^^6^ Its goal is to predict a 10-year risk of fractures associated with osteoporosis. Its algorithm is based on the individual analysis of each patient, correlating the risk factors: age, sex, Body Mass Index (BMI), history of bone fragility fractures, family history of hip fracture , smoking, prolonged use of corticosteroids, rheumatoid arthritis, other causes of secondary osteoporosis and high alcohol consumption. Such clinical data can be corroborated by the inclusion of Bone Densitometry (BD) results.^6^

The combination of risk factors and BD is optimal, but the latter may be considered only in targeted groups for purposes of rationalization and access to health services and resources.^1^^,^^5^^,^^7^ On the basis of population-based studies, it is plausible to suggest the initial assessment of fracture risk using FRAX without BD score and, in individuals at intermediary or high risk, to perform bone mineral density measurement using dual-energy X-Ray absorptiometry followed by the reassessment of the risk with FRAX including BD.^5^

According to this proposal, the assessment and intervention thresholds may be estimated, and a cost-effective diagnostic or therapeutic measure might be implemented.^5^ The use of the FRAX tool without the inclusion of data from bone densitometry allows treatment to be started for patients at high risk of fracture before densitometry is performed.^8^ There is good evidence that the FRAX score without the information on bone mineral density not only efficiently identifies patients at higher risk, but also reduces the unnecessary use of resources related to the BD exam,^9^ with suggestions to use this tool as a strategy to rationalize the request for BD in contexts of reduced resources.^10^ This has also been considered as a rational screening strategy in primary care.^11^^,^^12^

Although some studies show a good correlation between the estimated fracture risk without and with densitometric data,^7^ others have indicated an underestimation of the fracture risk when BD score is not included in the calculation,^13^^,^^14^ and there are contradictory results to establish the FRAX tool without the densitometry data as the preferred screening strategy.^15^

Considering that clinical factors influence the risk of fracture and that the FRAX tool in its Brazilian version considered specific epidemiological data from the country's population, using the FRAX tool and its fracture risk calculator to rationalize access to BD seems essential both to an individual level and in public health planning. In this sense, the authors consider studies that investigate the performance of FRAX calculations with and without BD in clinical settings are relevant and represent a reliable way to address the effectiveness of the exam to define the therapeutic management and screening algorithms.

The primary objective of this study is to describe and perform comparisons between the performance of the estimated risk of fractures in 10 years using the FRAX calculator based on clinical factors with and without BD results for women over 40 years of age with clinical diseases monitored in a tertiary care service in internal medicine.

## Methods

Observational cross-sectional study: after approval by the institutional research ethics committee (CAAE 39637720.1.0000.0068), a review of medical records was initiated to calculate the estimated risks of major and hip fractures using only clinical information followed by new calculation using clinical information plus bone mineral density results.

Inclusion criteria: the review was done on medical records of women aged 40 years or older with scheduled appointments at the internal medicine outpatient clinic of a university tertiary hospital between September 3^th^ and November 11^th^ 2020. During this period, 1935 medical appointments were scheduled at the present study's service. Of these, 1240 (64%) were women, and 1182 (95.3% of all women) corresponded to women 40 years of age or older. The medical charts from the 1182 women equal or over 40 years old were reviewed. The records included in the study were those of patients that underwent bone mineral density measurement using dual-energy X-Ray absorptiometry requested by their usual care team within 24 months prior to the present appointment. All exams were performed in the radiology department of the same tertiary hospital. Two subsets of data were created according to the reason for BD ordering: osteoporosis treatment or screening.

### Data acquisition

Medical record review with extraction of the following information: age, weight, height, family history (parents) of hip fracture, current smoking, glucocorticoids, rheumatoid arthritis, causes of secondary osteoporosis, alcohol consumption, femur T-score.

Risk estimations: the 10-year risk of fractures was estimated by the FRAX-BRAZIL (www.sheffield.ac.uk/FRAX/tool.aspx) with (FRAX BDI) and without (FRAX BDNI) the inclusion of the T-score. Calculation of normal and intervention thresholds by sex and age considered women from 40 thru 90 years old, and BMI of 25 kg/m^2^ without and with a previous fragility fracture, respectively;1 The individual risk for major and hip fractures was classified as low (at or below the normal threshold for age and sex), intermediate (between the normal and the intervention thresholds) or high (above the intervention threshold). Cases with different classifications based on FRAX score with and without the inclusion of BD were identified and analyzed.

To assess multimorbidity, two indices associated with clinical prognosis and mortality were used, the Elixhauser Comorbidity Index^16^ and the Charlson Comorbidity Index.^17^

### Statistical analysis

Shapiro-Wilk test to assess data normality; Wilcoxon signed-rank test for comparison of two related scores; Spearman's correlation coefficient to assess the correlation between FRAX results with and without BD. Bland-Altman analysis and plot were performed for agreement assessment. The level of statistical significance adopted was up to 1% (p-value <0.01). Statistical analysis was performed using GraphPad Prism version 9.0 for Windows, GraphPad Software, San Diego, California USA, www.graphpad.com.

## Results

Among the 1182 patients whose medical records were evaluated, 239 (20.2%) had undergone bone densitometry within 24 months from the moment of the data acquisition and had all the needed information to calculate FRAX estimates and were included. Data obtained from medical records referring to 239 women were analyzed (Table 1). The mean age was 65±10.35 years. Mean Body Mass Index (BMI) was 29.68±6.27 kg/m^2^. Considering the risk factors included in the FRAX tool: 32 (13.4%) patients had previous fractures; 23 (9.6%) current smoking; 78 (32.6%) corticosteroids use; 44 (18.4%) rheumatoid arthritis; 38 (15.9%) had secondary causes of osteoporosis; none had alcohol consumption greater than 3 doses a day, and no patient had reported a family history of fracture in her medical record (however, only a few had well-documented negative information.

**Table 1:** Characteristics of participants.

**Age** (mean ± SD)	65 ± 10.35 years
**Body mass index** (mean ± SD)	29.68 ± 6.27 kg/m^2^
**T-score** (mean ± SD)	1.33 ± 1.41

FRAX BRAZIL was calculated without the inclusion of BD (FRAX BDNI) and with BD included (FRAX-BDI) in order to estimate the 10-year risk of major bones and hip fractures. Calculated FRAX scores distributed in a right-skewed, not normal curve. A nonparametric Wilcoxon test was applied to compare paired FRAX scores (with and without BD) revealed statistically significant higher values of risk of fracture when BD was not included in the FRAX equation, except for tests used to treatment control (Table 2).

**Table 2:** Summary of risk assessment tools with and without bone-density information for patients undergoing DEXA for screening or treatment control indications.

	Study population (n = 239)		Screening (n = 162)		Treatment (n = 77)	
Risk of major fractures	FRAX BRAZIL BDNI	FRAX BRAZIL BDI	FRAX BRAZIL BDNI	FRAX BRAZIL BDI	FRAX BRAZIL BDNI	FRAX BRAZIL BDI
Median % (IQR25-75)	5.1 (3.3‒8.4)	4.3 (3.0‒7.7)	4.3 (3.0‒7.8)	3.7 (2.7‒5.4)	7.2 (4.1‒12.0)	7.3 (4.5‒11.0)
Wilcoxon test (p-value)	<0.0001		<0.0001		0.40 (NS)	
Spearman correlation *r* (95% CI)	0.79 (0.74‒0.84)		0.79 (0.72‒0.84)		0.72 (0.58‒0.81)	
Bland-Altman Bias						
Mean ± SD	1.243 ± 0.5146		1.23 ± 2.63		0.61± 4.557	
95% Upper limit of agreement	2.252		6.386		9.548	
95% Lower limit of agreement	0.2343		-3.923		-8.317	
**Risk of Hip Fractures**	**FRAX BRAZIL BDNI**	**FRAX BRAZIL BDI**	**FRAX BRAZIL BDNI**	**FRAX BRAZIL BDI**	**FRAX BRAZIL BDNI**	**FRAX BRAZIL BDI**
Median % (IQR25-75)	1.3 (0.6‒3.6)	0.8 (0.2‒2.2)	1.0 (0.5‒2.6)	0.5 (0.1‒1.3)	2.6 (0.8‒5.2)	2.2 (0.8‒4.2)
Wilcoxon test (p-value)	<0.0001		<0.0001		0.15 (NS)	
Spearman correlation *r* (95% CI)	0.69 (0.62‒0.75)		0.66 (0.56‒0.74)		0.63 (0.47‒0.75)	
**Bland-Altman Bias**						
Mean ± SD	3.2 ± 4.898		0.9852 ± 2.150		0.687 ± 3.688	
95% Lower limit of agreement	-6.4		-3.23		-6.542	
95% Upper limit of agreement	12.8		5.2		7.916	

IQR, Interquartile Range; 95% CI, Confidence Interval 95%; BDI, Body Densitometry Included; BDNI, Body Densitometry Not Included; SD, Standard Deviation.

**Fig. 1:**
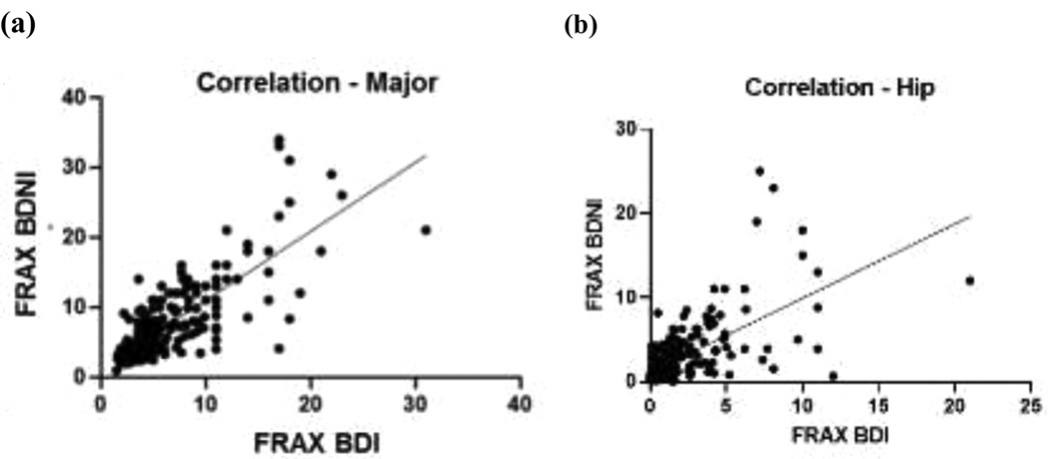
Correlation of Brazilian FRAX BDNI and BDI for major fractures and hip fractures. FRAX BDNI, Brazilian FRAX calculated with Bone-Density Not Included; FRAX BDI, Brazilian FRAX calculated with Bone-Density Included.

This was corroborated with the number of patients classified at low, intermediate, and high risk for major and hip fractures in 10 years (Table 3). Spearman correlation coefficients between FRAX without and with BD for major fractures shows *r* = 0.793 (95% CI 0.7388‒0.836) as shown in Fig. 1 and Table 2. For hip fractures 10-year risk, the correlation coefficient was *r* = 0.6922 (95% CI 0.6174‒0.75446).

**Table 3:** Patients classification by risk of fracture in 10 years.

		Women in risk of fracture in 10 years (n)		
Major Fractures		Low	Intermediate	HIGH
Total (n = 239)	FRAX BDNI	82 (34%)	93 (39%)	64 (27%)
	FRAX BD	101 (42%)	89 (37%)	49 (21%)
**Hip Fractures**				
Total (n = 239)	FRAX BDNI	77 (32%)	89 (37%)	73 (31%)
	FRAX BD	140 (59%)	56 (23%)	43 (18%)

BDI, Body Densitometry Included; BDNI, Body Densitometry Not Included.

**Fig. 2:**
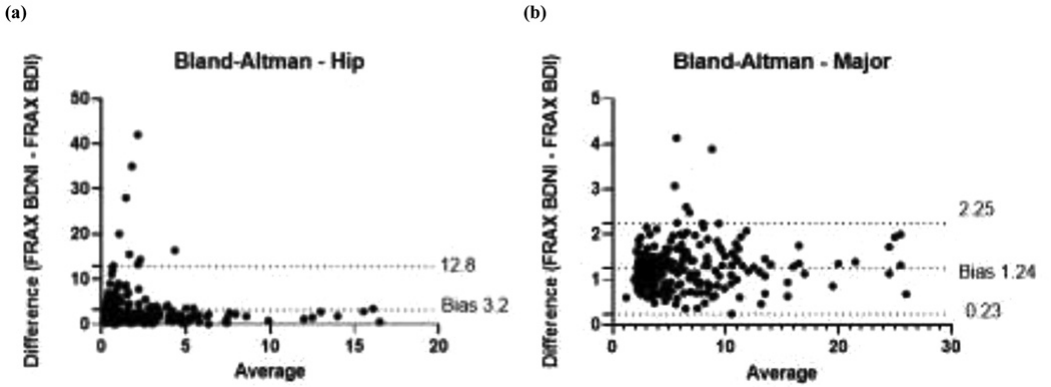
Bland-Altman analysis for concordance between methods, comparing FRAX calculated with bone density not-included (FRAX BDNI) and FRAX calculated with bone-density included (FRAX BDI).

The data were then separated in two subsets: one obtained from patients who underwent densitometry as a screening test (named "Screening"; n = 162; 67.8%) and another one in which the examination was indicated for treatment control (named "Treatment"; n = 77; 32.2%). For each subset, it was calculated the median and interquartile range, the p-value from nonparametric tests, and the correlation coefficients of FRAX-BDNI and FRAX-BDI, (Table 2). The full study population correlation curve is illustrated in Fig. 1a and b. Finally, the bias of overestimation or underestimation of risk induced by FRAX-BDNI in relation to the scores from FRAX-BDI, provided by Bland-Altman bias estimate, are presented in Table 1 and plotted in Fig. 2a and b. According to these, the FRAX-BDNI, for example, overestimated the 10-year risk of major fracture by a mean of 1.23±2.63% in relation to the scores of FRAX-BDI for patients in the Screening subset.

Bland-Altman analysis for major fractures shows a bias of 1.243±0.5146 comparing FRAX BDNI and FRAX BDI, demonstrating an excess of 1.243% in 10-year major fracture risk when using FRAX-BDNI (Fig. 2). The 95% limits of agreement are from 0.2343 to 2.252. In relation to the 10-year risk of hip fractures, Bland-Altman's analysis shows a bias of 3.2±4.898, with 95% limits of agreement from -6.4 to 12.8.

If NOGG^5^ recommendations to perform DEXA only in patients at intermediate risk by FRAX were followed, the authors would reduce the densitometry request for those at intermediate risk before deciding to start treatment. Only 62 of 168 (36.9%) patients would have undergone the examination for therapeutic definition, which would reduce costs and time to start treatment since 42 of these 168 (25%) patients would have received treatment directly before DEXA was performed, as they are at high risk of fracture. In other words, 38.1% of patients would be reassured of having a low risk of fractures and would not have the need to perform DEXA. After DEXA, 26 of those high-risk patients would have been reclassified and would not receive the recommendation to receive treatment. Three patients that were initially classified as low or intermediate risk would receive treatment. Thereby, DEXA was advantageous to define conduct in 17.26% of screening patients, being FRAX BDNI more sensitive than specific.

Regarding multimorbidity, mean Elixhauser comorbidity index was 4.12±1.88, being the 15 most prevalent diagnosis: systemic arterial hypertension (182 patients, 76.15%); diabetes (127 patients ‒ 53.23%, 66 without complications ‒ 27.61% and 61 with complications ‒ 25.52%); obesity (122 patients, 51%); collagen diseases and/ or rheumatoid arthritis (88 patients, 36.82%); hypothyroidism (71 patients, 29.7%); depression (71 patients); peripheric vascular disease (50 patients, 20.92%); chronic renal disease (49 patients, 20.5%); heart failure (31 patients, 12.97%); neurologic diseases (30 patients, 12.55%); chronic obstructive pulmonary disease (28 patients, 11.71%); solid tumor without metastasis (24 patients, 10.04%); blood loss anemia (24 patients); cardiac arrythmias (18 patients, 7.53%); pulmonary circulation disorders (17 patients, 7.11%), liver disease (17 patients). Mean Charlson Comorbidity index was 2.58±1.96.

There was no correlation between Elixhauser comorbidity index and FRAX scores in all the categories evaluated (rs = 0.00492, p = 0.93 for Elixhauser and FRAX-BDI for major fractures; rs = 0.044, p = 0.49 for Elixhauser and FRAX-BDI for hip fractures; rs = 0.045, p = 0.48 for Elixhauser and FRAX-BDNI for major fracures; rs = 0.074, p = 0.25 for Elixhauser and FRAX-BDNI for hip fractures).

## Discussion

The present study's findings suggest that using FRAX to estimate 10-year fracture risk without densitometric data might be a reliable tool for screening, even for patients with a high prevalence of risk factors, improving accessibility and equity in health systems. The present study's data suggest an overestimation of fracture risk using the methodology without BD, suggesting that it is safe to be widely used as a screening tool.

The presented results are relevant because they represent an assessment of the performance of the Brazilian version of FRAX in a setting of patients with high-complex diseases and multimorbidity, with a high prevalence of risk factors. There was a statistically significant overestimation of risks with FRAX without BD, but clinically this enhancement of risk may not be relevant, as the bias for screening patients was 1.23 (meaning a mean overestimation of 1.23% of the risk in 10-years); In the scope of the assessment of FRAX BDNI for screening purposes, it is possible that the overestimation of risk-based upon clinical factors only could help to improve its sensibility.

The correlation of FRAX BDNI and FRAX BD is better for major bone fracture risk than for hip fracture risk, and the difference between FRAX BDNI and FRAX BD for patients under treatment is even smaller.

In previous studies, there are conflicting results related to the correlation between the estimated fracture risk without and with densitometric data,^7^ with some evidence of underestimation of the fracture risk with this methodology.^13^^,^^14^ In the comparison between the FRAX tool with and without the inclusion of densitometric data for the definition of therapeutic conduct in a population composed of 119 men, the use of the FRAX tool without densitometric data was as effective as the use of FRAX with densitometric data in what it concerns both the prediction of the risk of fragility fractures and the therapeutic suggestions derived from its application.^18^ In the evaluation of 151 patients, 84% of them had an agreement of the risk assessed with and without the complimentary exam,^19^ results that are corroborated by other authors in other populations.^20^ In India, the agreement was similar (86.6%), and the authors suggest using this tool as a strategy to rationalize the request for bone densitometry in contexts of reduced resources,^10^ as well as being considered as a screening strategy in primary care.^11^^,^^12^ There is evidence that the FRAX score without the information on bone mineral density not only efficiently identifies patients at higher risk, but also reduces the unnecessary use of resources related to the bone densitometry exam,^9^ including cohorts that followed the patients over the 10-year period for which FRAX estimates the risk.^21^ In contrast, in a Japanese study that included 13421 participants, the sensitivity of using the FRAX tool without the densitometric data was not sufficient to use this strategy as a screening.^15^

Overdiagnosis and overtreatment of osteoporosis have been under discussion,^22^, ^23^, ^24^ and an agenda of studies and guidelines that weight risks and benefits must be organized in order to clarify the most useful framework for screening and therapeutic decision and in this way, the present study would be helpful as it addresses this question on a population that is multimorbid. Still, strategies that promote rational osteoporosis screening and diagnosis are welcome in order to improve clinical outcomes and favor equity in health systems, such as quantitative ultrasound sonography^25^ and other risk assessment tools.^26^ The cost-effectiveness of osteoporosis screening is a relevant target of research, and the current study provides reflection as 64 of 168 screening bone-density exams might not be necessary.

Socioeconomic and populational diversity are important factors for osteoporosis burden, making it important that country and ethnic specificities are addressed in studies.^26^ As a population derived from a tertiary hospital, the study's patients show a high prevalence of multimorbidity,^27^ which may exert independent effects on fracture risk, as well as other risk factors (e.g. another endocrine, or inflammatory disease, fall risk, and use of medications) that are not acknowledged in FRAX.^1^^,^^28^ However, in the present study's patients, there was no correlation between FRAX scores and Elixhauser comorbidity index, suggesting that multimorbidity did not influence fracture risk assessment using FRAX in this population, thus making the authors’ findings generalizable for other outpatient settings that are less complex.

Further refinement targeted to selected cases in which adding BD score to FRAX equation would possibly improve risk grading with positive consequences on clinical decisions and endpoints is desirable. Additionally, clinical endpoints were not evaluated. So, it is imperative that the 10-year incidence of fractures should be evaluated in this real-life scenario as a cohort. Further studies are also needed to address the 5% of patients who did not receive an indication of treatment initially but should have received after reassessment with BMD data.

## Conclusion

In conclusion, this study suggests that estimating the 10-year risk of fragility fractures using FRAX without BD is a reliable and sensitive tool in comparison to the estimated 10-year risk of fragility fractures using FRAX with BD; and thus, the authors advocate it as a screening tool, even in high-risk, multimorbid contexts.

## Authors' contributions

Favarato MHS: Article conception, data collection and analysis, manuscript writing and revision, and figures preparation.

Almeida MF: Data collection and manuscript revision.

Lichtenstein A: Critical discussion of findings, and manuscript review.

Martins MA: Critical discussion of findings, and manuscript review.

Ferreira Junior M: Article conception, data analysis, tables preparation, and manuscript writing and revision.

## Conflicts of interest

The authors declare no conflicts of interest.
